# Implementation of Chaotic Synchronization and Artificial Neural Networks in Modified OTP Scheme for Image Encryption

**DOI:** 10.3390/jimaging11040121

**Published:** 2025-04-17

**Authors:** Hristina Stoycheva, Georgi Mihalev, Stanimir Sadinov, Krasen Angelov

**Affiliations:** 1 Department of Communication Technique and Technologies, Faculty of Electronics and Engineering, Technical University of Gabrovo, 5300 Gabrovo, Bulgaria; murry@tugab.bg (S.S.); kkangelov@tugab.bg (K.A.); 2 Department of Automation, Information and Control Systems, Faculty of Electronics and Engineering, Technical University of Gabrovo, 5300 Gabrovo, Bulgaria; gmihalev@tugab.bg

**Keywords:** image encryption, secure communication, chaos, chaotic synchronization, artificial neuron network, OTP algorithm

## Abstract

This paper presents a modified image encryption scheme based on the OTP (One-Time Pad) algorithm, consisting of chaotic synchronization and artificial neural networks (ANNs) for improved security and efficiency. The scheme uses chaotic synchronization based on feedback control to create complex and unique encryption keys. Additionally, ANNs are used to approximate time functions, creating a neural encoding key, which adds an additional layer of complexity to the encryption process. The proposed scheme integrates static, chaotic, and neural keys in a multilayer structure, providing high resistance against statistical and cryptographic attacks. The results show that the proposed methodology achieves entropy values close to the theoretical maximum, effectively destroys the correlation between pixels, and demonstrates high sensitivity to variations in the input data. The proposed scheme shows very good feasibility in terms of both security and efficiency, which gives a reliable solution for secure image transmission and storage. This is proven by a study of resistance to various crypto–graphic attacks such as brute force attack, differential attack, noise and data cut attacks, key sensitivity, and computational complexity.

## 1. Introduction

Digital images are the most common multimedia used for both communication and entertainment. In everyday life, people encounter digital information not only on social networks, most of which are based on published images or videos, but also in modern means of communication, such as Zoom, Google Meet, Mesenger, WhatsApp, etc. To a large extent, journalistic and lifestyle publications are presented through digital formats, which significantly increases their audience. And while all these platforms, applications, and sites rely on built-in protections and good intentions on the part of users, the issue of transmitting sensitive digital information through unprotected communicational networks is not so simple.

In communication networks where security is of paramount importance, the secure transmission of sensitive information can be roughly divided into two directions: encryption of the communication channel or encryption of the information itself. The second direction is a more common object of research that considers the encryption of the sensitive information itself and its subsequent transmission over public unprotected communication networks. When this sensitive information is a digital image, the main problem is the implementation of such an encryption scheme that gives satisfactory results not only in terms of encryption level, but also in terms of reliability and speed, with minimal losses throughout recovery.

In the scientific literature of the last decade, a variety of approaches to image encryption have been observed, with researchers combining different techniques and developing new algorithms to enhance encryption security and efficiency. The main categories and commonly used algorithms include the following: Symmetric cryptographic algorithms, including block ciphers such as AES (Advanced Encryption Standard) [1,2] and stream ciphers such as Rivest Cipher 4 [3,4]; asymmetric cryptographic algorithms, such as ECC (Elliptic Curve Cryptography), which offers shorter keys while maintaining the same level of security, making it attractive for resource-constrained applications [5,6]; permutation- and diffusion-based algorithms, which aim to disrupt the statistical structure of the image, making it unrecognizable [7,8]; and quantum-resistant algorithms [9,10,11,12], among others.

**Literature Review:** Three main directions in the encryption algorithms that are subject to active research and modification can be defined: innovative—such as the encryption algorithms based on artificial neural networks (ANNs); fundamental—the most common among them is the One-Time Pad (OTP) algorithm; and eccentric—which is where chaos-based encryption algorithms fall.

**ANN in encryption algorithms:** ANN-based encryption algorithms [13,14,15,16] are an innovative approach that combines the power of machine learning with cryptography. ANNs can be trained to perform cryptographic tasks such as transforming data into ciphertext [17], are used to generate pseudorandom keys that are difficult to predict [18], and can create complex cryptographic schemes [19]. Among the advantages of ANN encryption algorithms are self-adaptability, resistance to traditional attacks, generation of personalized cryptographic schemes for specific applications, and speed of operation (after going through the training process). The disadvantages of this type of encryption methods include the requirement for large volumes of training data, high computational complexity, vulnerability to specific attacks (such as those that exploit their structure), and some difficulties related to security verification.

**One-Time Pad based algorithms:** One of the fundamental algorithms for image encryption is the One-Time Pad (OTP) algorithm. It is widely used due to its easy implementation and high level of security [20,21,22,23,24]. In this case, if the key is completely random, equal in length to the message (or image), and is never reused, OTP provides theoretically perfect security. In image encryption, OTP algorithms are an encryption technique that requires the use of a one-time pre-shared key with a size equal to that of the image or larger. Encryption is performed by applying a bitwise XOR between the data and the key. This technique is one of the most elementary and generally represents pairing the image with the generated secret key. The algorithm is equivalent to the masking method in signal encryption. In this type of algorithm, to achieve a high level of security, the complexity of the generated key is relied on. The advantages of OTP algorithms for image encryption include high security and easy implementation. On the other hand, the following challenges associated with implementing OTP algorithms for image encryption should not be overlooked: the large key size; ensuring secure storage and transmission of the key; and achieving complete randomness of the key.

**Chaos in encryption algorithms:** There is a growing trend in the application of encryption algorithms based on chaotic systems. What makes chaos attractive for use as a basis for developing cryptosystems is mainly its random behavior, sensitivity to initial conditions and the set of parameters that meet the classical Shannon requirements for confusion and diffusion [25]. Algorithms based on chaos show some extremely good properties in many aspects in terms of security, complexity, speed, etc. Such algorithms can be based on the use of one-dimensional and two-dimensional chaotic maps—used to generate pseudorandom values that shift and modify pixels [26,27,28,29]; fractal chaotic maps—use fractal structures generated by chaos [30,31,32]; three-system chaotic maps—generate three chaotic sequences to encrypt the RGB components separately [33,34]; and adaptive chaotic algorithms, in which chaotic parameters are tuned based on the histogram or entropy of the input image [35,36], among others [37,38,39,40,41,42]. The advantages of chaos-based image encryption algorithms are high security due to the complex dynamics and sensitivity to the initial conditions and parameters of chaotic systems; low computational cost; and flexibility—they are easily adapted for different types of images.

The choice of an appropriate algorithm depends on the specific requirements of the application, including the level of security, computational resources and resistance to various types of attacks. This is precisely what determines the combination of more than one approach to image encryption and the construction of new schemes for the secure transmission of sensitive information via unprotected communicational networks.

**Contribution:** This article proposes a modification of an image encoding scheme based on a standard OTP algorithm, modified by implementing a multilayer structure with the implementation of a chaotic signal and data from the OTP approximating various encoding functions. By implementing a subsequent chaotic synchronization scheme, the recovery of the transmitted encoded image is achieved. Key points of the proposed modification are the higher degree of reliability achieved by forming a unique encryption key that is not repeated and is not used repeatedly, making the OTP scheme practically unbreakable; the use of chaotic synchronization based on feedback control leads to high quality image recovery; and the used basic structure of the OTP algorithms preserves the efficiency of the subsequent operations of compression, transfer, and storage of images.

The main goals of the developed scheme are to achieve a high degree of security and efficiency in encoding while preserving the quality of the restored images during decoding.

**Our main contributions are as follows:**

- This article proposes an innovative multilayer cryptographic scheme based on the OTP algorithm, integrating static, chaotic, and neural encoding keys with the aim of enhancing security and protection against cryptographic attacks.
- The proposed algorithm implements chaotic synchronization of two identical Chua chaotic systems through feedback control, which ensures reliable image encoding and decoding, and enables the concealment of the source of cryptographic information.
- An ANN generator has been implemented to provide an additional neural key based on the approximation of time-dependent functions. This reduces the influence of the human factor and adds complex dynamic behavior.
- In the encoding process, a scrambling-based encryption of the three-color channels is successfully implemented using the same chaotic signal that was used to generate the chaotic encoding key.
- Through simulations and statistical analysis of key metrics, the algorithm has been proven to be highly effective against various types of attacks.

The rest of this article is organized as follows: In Section 2, the basic method for implementing chaotic synchronization, through feedback control with one-way coupling, the ANN apparatus for approximating temporal functions and classical approaches for analyzing the coding rate and image reconstruction quality are presented. In Section 3, the proposed modified OTP image coding scheme is described in detail. In Section 4, analytical and simulation results for coding different images using the proposed approach with the implementation of a synchronization scheme using FFNN for approximating three types of functions are presented. The conclusion and inferences from the obtained results are presented in the last section.

## 2. Preliminaries

Image encoding mainly aims at a high level of protection, quality preservation, data compression, efficiency preservation during transfer and storage, etc. The use of advanced security mechanisms and intelligent tools is essential to achieve these goals and maintain reliability. The current Section presents a detailed presentation of the methods and techniques used to implement a modified image encoding scheme

### 2.1. Synchronization of Chaotic Systems Based on Feedback Control

Most of the chaos-based communication systems are based on synchronized chaotic systems. This means that under certain circumstances the complex and highly sensitive nonlinear dynamics of the coupled chaotic systems are synchronized and the resulting synchronous state can be used in the communication scheme in several different ways.

One of the remarkable features of chaotic communication is the possibility of implementation at the physical level. From the point of view of cryptography, chaos has many desirable properties, namely complexity, ergodicity, transitivity, determinism and sensitivity to initial conditions and perturbations [43]. Cryptography is also the topic that receives the majority of publications in the field of chaos applications.

Chaos is particularly attractive for use as a basis for the development of cryptosystems due to its randomness, strong sensitivity to initial conditions and parameters, which fully satisfy the classical criteria for confusion and diffusion [44]. Chaos-based algorithms exhibit some exceptionally good properties in many aspects in terms of security, complexity, speed, etc.

Considering that chaotic systems are inherently nonlinear systems, some of the established theories and methods for controlling nonlinear systems can be used in the synchronization of chaotic systems. The chaotic systems under consideration are of a type that allows them to be described by ordinary differential and difference equations.

The synchronization method used is based on feedback control, i.e., an additional signal proportional to the mismatch is fed to one of the systems. Avoiding the strict mathematical description of the method presented in [45], we choose to refer to the lighter algorithmic interpretation.

Let’s consider a nonlinear SISO system in the following simplified form:

(1) $$\dot{x}=f\left(x\right)+g\left(x\right)u\;y=h(x)$$

where $\dot{x}\in R^{n}$ is the state vector, $u\in R^{p}$ is a vector of inputs, and $y\in R^{m}$ is the vector of outputs.

The relative degree of the system r at the point x_0_ is determined, if

(2) $$L_{g}L_{f}^{k}h\left(x\right)=0,\;за\;0\leq k<r-1,\;\forall x\in U\;L_{g}L_{f}^{r-1}h\left(x\right)\neq 0$$

If the system has relative degree r ≤ n, where n is the order of the system (1), the coordinate transformation functions are formed:

(3) $$S_{1}\left(x\right)=h\left(x\right)\;S_{2}\left(x\right)=L_{f}h(x)\;\vdots \;S_{r}\left(x\right)=L_{f}^{r-1}h(x),$$

by forming a mapping of the type

(4) $$\;S(x)=\left(\begin{array}{c} S_{1}(x)\\ \vdots \\ S_{r}(x) \end{array}\right).$$

If r < n, it is always possible to find n-r additional functions, such that the transforming map (4) has a Jacobian matrix of full rank at x_0_, under the additional condition that $L_{g}S_{i}(x)=0$, for all r + 1 ≤ i < n and for ∀x in a neighborhood of x_0_. In this case, the additional equations to the dynamics of the system are defined by

(5) $$\;q_{i}(z)=L_{f}S_{i}(S^{-1}(z))\;\mathrm{for}\mathrm{all}r+1\;\leq \;i<n$$

By means of the transformation z = S(x) from (4) and provided that the system (1) has a relative degree r at x_0_, it can be transformed into

(6) $${\dot{z}}_{1}=S_{2}\left(x\left(t\right)\right)=z_{2}\left(t\right)\;{\dot{z}}_{2}=S_{3}\left(x\left(t\right)\right)=z_{3}(t)\;\vdots \;{\dot{z}}_{r-1}=S_{r}\left(x\left(t\right)\right)=z_{r}\left(t\right)\;{\dot{z}}_{r}=b\left(z\right)+a\left(z\right)u\;{\dot{z}}_{r+1}=q_{r+1}(z)\;\vdots \;{\dot{z}}_{n}=q_{n}(z)\;y=z_{1}$$

At r = n, a(z) ≠ 0, for each $S^{-1}(z)\in U(x_{0})$, the new input will be

(7) $$u=\frac{1}{L_{g}L_{f}^{r-1}h\left(x\right)}(v-L_{f}^{r}h(x)),$$

whereby system (6) will become a linear control system described as

(8) $${\dot{z}}_{1}=z_{2}\;{\dot{z}}_{2}=z_{3}\;\vdots \;{\dot{z}}_{n-1}=z_{n}\;{\dot{z}}_{n}=v\;y=z_{1}.$$

In the synchronization tasks of chaotic systems, a synchronization scheme is most often synthesized between two identical, continuous systems with a one-way connection between them.

**Figure 1:**
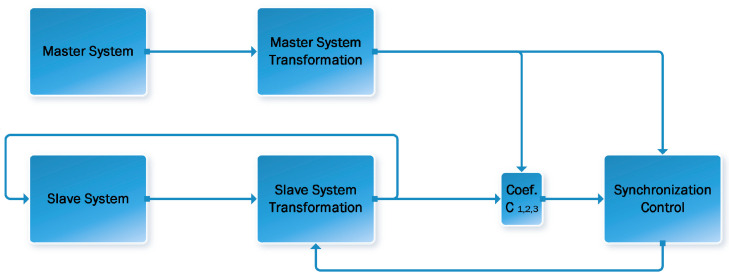
Generalized block diagram of the synchronization process.

The system that provides the synchronization signal is called the control system (9), and the one that receives this signal and adjusts its dynamics to that of the control system is called the controlled (slave) system (10), generalized block diagram of the synchronization process is presented on Figure 1. The two can be represented by the following generalized notation:

(9) $$Master:\;\left\{\begin{array}{c} \dot{x}=f\left(x\right),\\ y=h(x) \end{array}\right.$$

(10) $$\mathrm{Slave}\text{:}\;\left\{\begin{array}{c} \dot{\tilde{x}}=f\left(\tilde{x}\right)+g\left(\tilde{x}\right)u,\\ y=h\left(\tilde{x}\right)\;\;\;\;\;\;\;\;\;\;\;\;\;\;\;\; \end{array}\right.$$

Setting $\tilde{z}=S(\tilde{x})$ defined by (4), if there is a relative degree r (r ≤ n), the system (10) presented in a more compressed form can be transformed into

(11) $$\left\{\begin{array}{c} {\dot{\tilde{z}}}^{1}=A\tilde{z}+E(b(\tilde{z})+a(\tilde{z})u),\\ {\dot{\tilde{z}}}^{2}=Q\left({\tilde{z}}^{1},{\tilde{z}}^{2}\right)\;\;\;\;\;\;\;\;\;\;\;\;\;\;\;\;\;\;\;\;\;\;\;\;\;\;\;\;\\ \tilde{y}={\tilde{z}}_{1},\;\;\;\;\;\;\;\;\;\;\;\;\;\;\;\;\;\;\;\;\;\;\;\;\;\;\;\;\;\;\;\;\;\;\;\;\;\;\;\;\;\; \end{array}\right.\;$$

where A_rxr_ has the canonical form of Brunowski [46], $E={\left(0,0,\ldots ,1\right)}_{rx1}^{T}$, $a(\tilde{z})$ and $b(\tilde{z})$ are smooth mappings that can be determined using (6) and the new input v from (7). System (9) is transformed similarly.

After transforming the master and slave systems, the disagreement system is defined by $e={\left(e^{1},e^{2}\right)}^{T}={\left({\tilde{z}}^{1}-z^{1},{\tilde{z}}^{2}-z^{2}\right)}^{T}$, as

(12) $$\left\{\begin{array}{c} {\dot{e}}^{1}=Ae^{1}+E(b({\tilde{z}}^{1},{\tilde{z}}^{2})+a({\tilde{z}}^{1},{\tilde{z}}^{2})u-b(z^{1},z^{2})\\ {\dot{e}}^{2}=Q\left({\tilde{z}}^{1},{\tilde{z}}^{2}\right)-Q\left(z^{1},z^{2}\right),\;\;\;\;\;\;\;\;\;\;\;\;\;\;\;\;\;\;\;\;\;\;\;\;\;\;\;\;\;\;\;\;\;\;\;\;\;\;\;\; \end{array}\right.\;$$

with control that makes (12) asymptotically stable, defined by the following formula:

(13) $$u=-\frac{1}{a\left({\tilde{z}}^{1},{\tilde{z}}^{2}\right)}\left(b\left({\tilde{z}}^{1},{\tilde{z}}^{2}\right)-b\left(z^{1},z^{2}\right)+Ce\right).\;$$

Through the synthesized control, it is guaranteed that systems (9) and (10) can be synchronized asymptotically, i.e., $\underset{t\to \infty }{\lim}e_{i}\left(t\right)=0$.

### 2.2. Artificial Neural Network for Time Function Approximation

The second main element in the developed image coding scheme is the integration of the ANN. As already mentioned, the neural network aims at a more complex multilayer coding based on the OTP algorithm. The main idea behind the use of ANNs is to hide the original source of obtaining the coding key. ANNs are applied to solve problems of a diverse nature, such as classifiers, associative memories, simulators, approximators, etc. In the current implementation, the main task of ANNs is the approximation of time functions and subsequent generation of a coding key in the form of an image.

It is known that ANNs give very good results in approximating functions. The submission of values from numerical series as a data set is more inherent to artificial networks of the FFNN type, such as the ANN used in the current case.

The used multilayer neural network has one input and one output, with connections between neurons only in adjacent layers. There are two hidden layers between the input and output of the network, containing computational units, respectively 10 neurons in the first and 20 neurons in the second. The choice of the number of hidden layers and the neurons in them is based on Stone’s theorem and a generalization presented in [47]. The input-output relationship of each neuron is described by an input x_i_, an output y, connection weights w_i_, a threshold value θ and a differentiable function ϕ.

(14) $$y=\phi \left(\sum_{i=1}^{k}w_{i}x_{i}-\theta \right)$$

**Figure 2:**
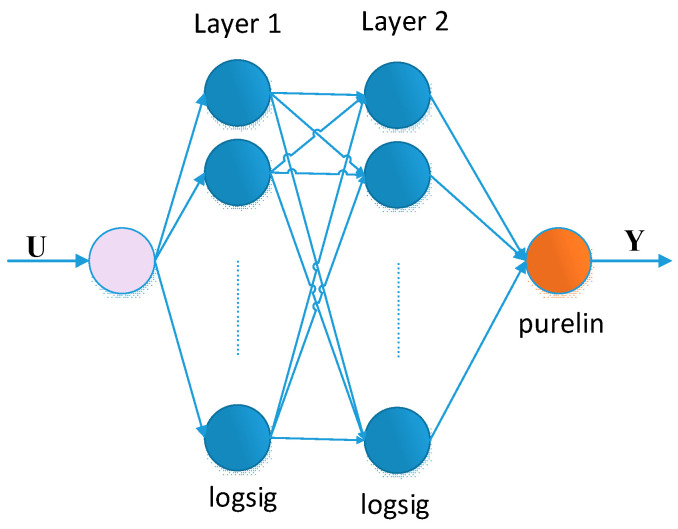
Artificial neural network of type 1/10/20/1.

Figure 2 shows the architecture of the neural network used in this encrypting scheme.

The network is trained using the Levenberg-Marquardt backpropagation method. This method is considered the fastest, since its efficiency is mainly influenced by the rational choice of the regularization factor used to calculate the updated term of the parameter vector w_i_ of the neural network. The regularization factor is functionally related to the gradient of the sum of the mean square errors, which allows us to overcome the limitation of the single-output ANN algorithm and makes it effective for function approximation. In terms of memory, this training algorithm requires memory of the order of O(w^2^), where w is the number of weight coefficients in the network.

**Figure 3:**
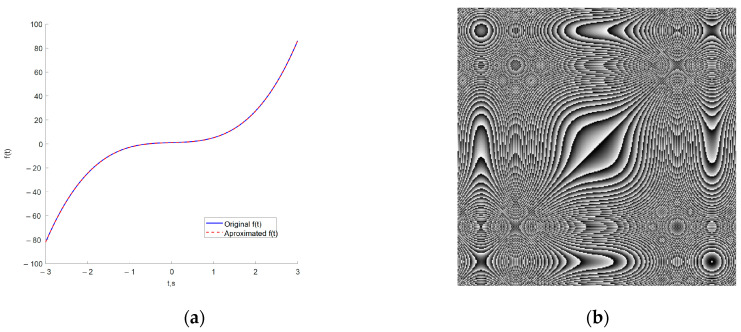
Approximation of a sample temporal function and formation of a coding image: (**a**) original and ANN-approximated temporal function; (**b**) formed coding image.

The graphical results of approximating the function $f\left(t\right)=3t^{3}+0.1t^{2}+t+1$ and the resulting image, which will be used to form an encryption key are shown in Figure 3.

From the images shown in Figure 3, it can be seen that the used ANN copes with the task of approximating complex time characteristics, as the achieved mean square error (MSE) is of the order of 1 × 10^−7^.

### 2.3. Methods for Statistical Analysis in Image Encryption

Regardless of the chosen image encryption and recovery process, the final assessment of whether a given image is highly encrypted or of good quality after recovery is based on the subjective judgment of the person. This is an essential and key point in the process of encoding/decoding digital images. For this reason, the analysis of the results of the decryption process is carried out based on three main methods, widely used in publications on the subject, namely histograms, correlations and information entropy.

#### 2.3.1. Histogram

The histogram measures the quality of encryption in terms of how it maximizes the difference between the original and encrypted images.

An image histogram uses a bar chart that shows the distribution of pixel brightness values. For greyscale images, the horizontal axis represents the grey level value, starting from zero and going up to 255. Each vertical column represents the number of occurrences of the corresponding grey level in the image. For image encryption algorithms, the histogram of the encrypted image must have two properties: it must be completely different from the histogram of the original image; it must have a uniform distribution, meaning that the probability of occurrence of any value in the grey scale is approximately the same.

A high level of image encryption is evidenced by a stable and smooth histogram of the encrypted image, while the number of occurrences of the corresponding grey level in the original image is unstable. This means that the resulting encrypted image does not provide any statistical information about the corresponding grey level.

#### 2.3.2. Correlation

Correlation analysis is a statistical data processing method used to study the coefficients (correlations) between variables. The analysis compares the correlation coefficients between one or more pairs of variables to establish statistical interdependencies between them. A useful indicator for assessing the quality of encryption of a given image is the correlation coefficient between pixels at the same indices in the original and encrypted images. This indicator can be calculated as follows:

(15) $$r_{xy}=\frac{cov(x,y)}{\sqrt{D(x)}\sqrt{D(y)}}$$

where x и y are the original and encrypted images. In numerical calculations, the following discrete formulas can be used:

(16) $$E\left(x\right)=\frac{1}{L}\sum_{l=1}^{L}x_{l}$$

(17) $$D\left(x\right)=\frac{1}{L}\sum_{l=1}^{L}{\left(x_{l}-E\left(x\right)\right)}^{2}$$

(18) $$cov\left(x,y\right)=\frac{1}{L}\sum_{l=1}^{L}\left(x_{l}-E\left(x\right)\right)\left(y_{l}-E\left(y\right)\right)$$

where L is the number of pixels involved in the calculations. The closer the value of $r_{xy}$ is to zero, the lower the information dependence between two neighboring pixels, and therefore, the better the quality of the encryption algorithm will be.

The graphical interpretation of the correlation of the encoded image should show a significant elimination of correlated pixels in the original image, which are usually located in the diagonal, vertical or horizontal direction. The purpose of the correlation pattern analysis is to identify the degree of connectivity between the original and encrypted images.

#### 2.3.3. Entropy

Entropy is a physical quantity that is a measure of disorder. Information entropy, originally proposed by Shannon, is one of the key measures for quantifying the degree of uncertainty (randomness) of a system with respect to information [25]. It can be applied to measure randomness in an image encryption system. Given an 8-bit grayscale level that has 256 possible pixel values, i.e., 0, 1, …, 255, the information entropy—H, can be formulated as the following equation:

(19) $$H\left(x\right)=-\sum_{i=0}^{255}p(x_{i})\log_{2}p(x_{i})$$

where $p(x_{i})$ is the probability with which the i-th gray value $x_{i}$ appears in image x. For an encrypted image, when each gray value $x_{i}$ appears with equal probability, i.e., $\frac{1}{256}$, the information entropy reaches a maximum of—8. Therefore, an ideal approach to encrypt images should have an information entropy close to 8.

## 3. Modified OTP Scheme for Image Encryption

When encoding an image, OTP algorithms are an encoding technique that must be based on a one-time pre-shared key with a size equal to or larger than that image. This technique is one of the most elementary and, in general, represents pairing the image with the created secret key, which provides great opportunities for implementing and exploring various additions and modifications. The algorithm is equivalent to the masking method in signal encoding. In this type of algorithms, to achieve a high level of protection, the complexity of the generated key is relied on. For this reason, the use of a chaotic signal and the implementation of chaotic synchronized schemes is very appropriate, the essence of the method. In the current implementation, the chaotic signal from the control system is used to increase a second additional key, which is combined with a pre-increased primary key of a pseudo-random nature. In addition, the chaotic signal is used to implement the scrambling process, individual for each of the color channels.

The use of chaotic signals in this type of algorithms is very valuable, since it achieves a condition for a one-time increase in the key, as well as achieving its high degree of complexity. In view of the international trends in improving ANNs and AI, an opportunity opens up to achieve even more complex coding by implementing a multilayer scheme. This multilayer is achieved by solving the fundamental task for the ANN apparatus—namely, approximating functions or signals. These functions are used to generate an additional coding key, which will be called a neural key from now on. The main idea of using ANNs is to hide the original source for obtaining the neural key.

To implement the modified OTP (One-time path) image encryption scheme, a chaotic synchronization scheme between two identical Chua chaotic systems is used according to the method shown in point 2.1.

**Figure 4:**
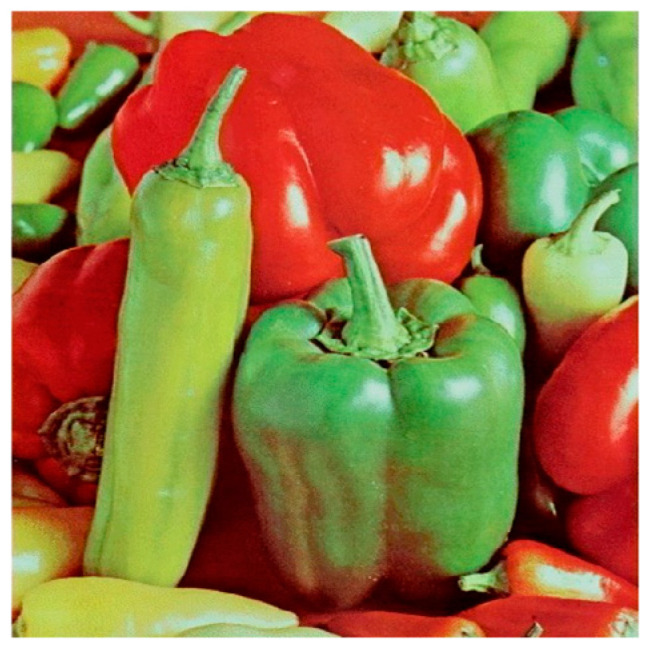
Original image.

When implementing the scheme, the following steps are followed:

**Step 1:** Representation of the input image (Figure 4)—A matrix interpretation of the input image is obtained by calculating its dimensions (rows and columns) and number of pixels.

**Figure 5:**
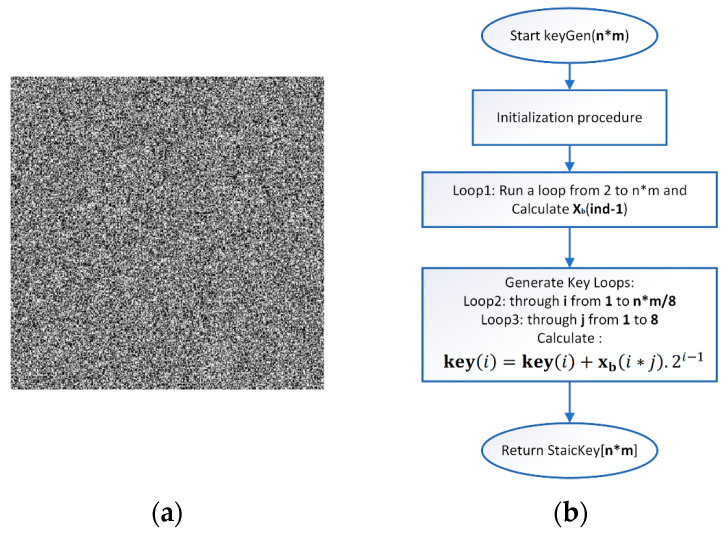
(**a**) Static key image; (**b**) Static key generation block diagram.

**Step 2:** Static key generation—The static key generation process is iterative, with the maximum number of iterations being n*m*8, where n and m describe the dimensions of the input image. A recursive algorithm is used to generate an array of numbers in the binary number system. The resulting array is converted to numbers in the decimal number system, reducing the size of the array by eight times. The extraction of each value from the key is performed using the following formula:

(20) $$key\left(i\right)=key\left(i\right)+x_{b}\left(i*j\right).2^{i-1},$$

where **key** is the array of the newly generated key with dimension n*m, $x_{b}$ is the array of binary numbers generated in the previous step, i varies in the ranges 1≤i≤n∗m, j varies in the range 1≤j≤8. Figure 5a presents the generated static key in the form of an image. Figure 5b presents a simple block diagram for the process of generating the Static key.

**Figure 6:**
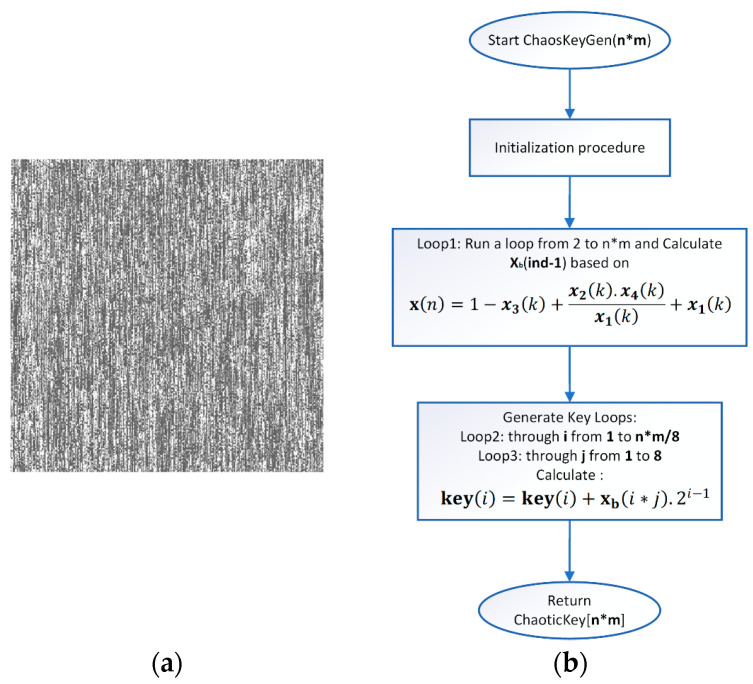
(**a**) Chaotic (dynamic) key; (**b**) chaotic key generation block diagram.

**Step 3:** Generate a chaotic (dynamic) key—The process of generating the second key is similar to the generation of the primary key described in the previous step. The difference occurs in the generation of the initial array, where each value is a function of the chaotic signal from the state vector of the chaotic control system and is calculated using the following formula:

(21) $$x\left(n\right)=1-x_{3}\left(k\right)+\frac{x_{2}\left(k\right).x_{4}\left(k\right)}{x_{1}\left(k\right)}+x_{1}\left(k\right),$$

where n and k are index variables, with k reflecting values of the chaotic signal after passing the transient process. In Formula (21) the use of $x_{4}\left(k\right)$ is intended to provide for the possibility of the algorithm to work with hyperchaotic systems of the 4th order. Since in the current implementation the system is of the third order, for the chaotic signal of the fourth state variable it is chosen to repeat that of $x_{3}$. After obtaining the primary array, the final one is obtained in an analogous way, i.e., by using Formula (21). Figure 6a shows the generated chaotic key in the form of an image. The chaotic key generation block diagram is presented in Figure 6b.

**Figure 7:**
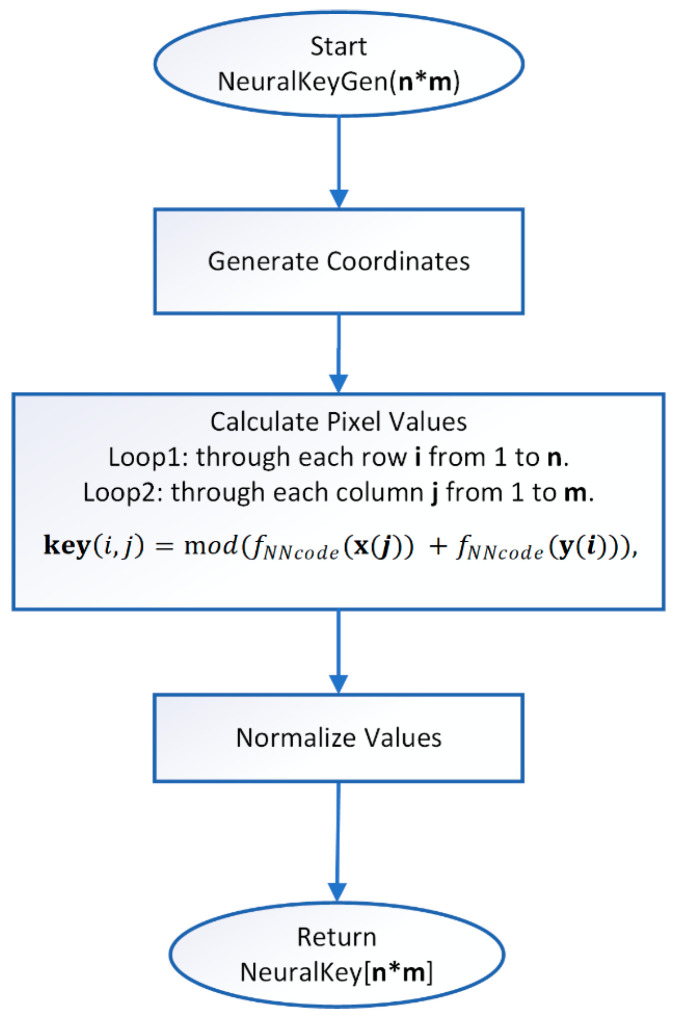
Neural key generation block diagram.

**Step 4:** Generation of a neural key—A similar iterative algorithm is used to generate the neural key, with the array of binary numbers being formed when generating a value of the function approximated by the ANN. The initial value can be chosen arbitrarily, but must be within the range of the function variation with which the training was conducted. When generating the encoding key, the x and y coordinates are generated, after which the image matrix is initialized. Each pixel of the generated image is formed using the following relationship:

(22) $$key\left(i,j\right)=mod(f_{NNcode}(x(j))+f_{NNcode}(y(i))),$$

where key is the matrix of the encoding image, $f_{NNcode}—$a value from the ANN approximating a given function, i and j—index variables, x(j) and y(i) are the horizontal and vertical coordinates of the matrix. The type of image obtained from a given function can be seen in Figure 3b in Section 2.2. Simple block diagram of the Neural key generation process is presented in Figure 7.

**Figure 8:**
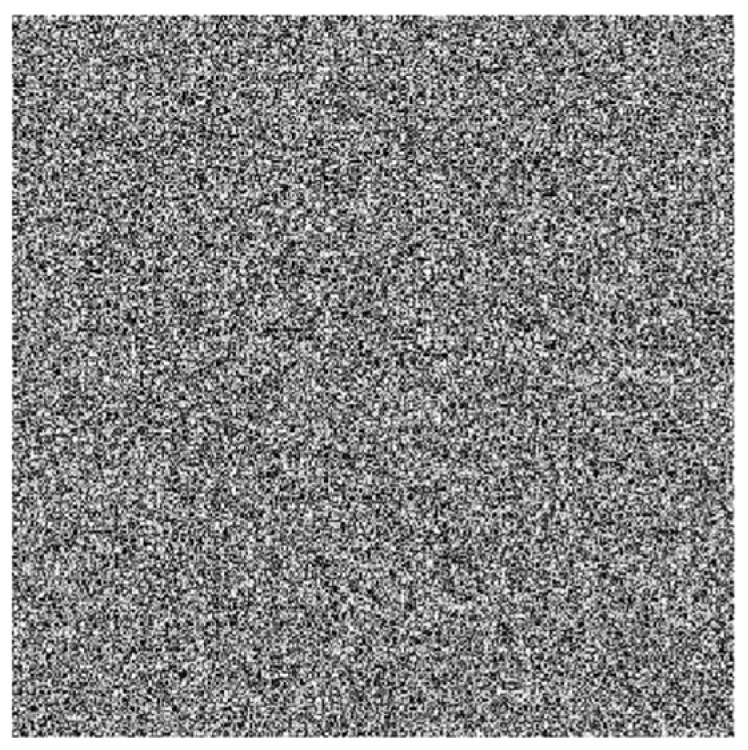
General chaotic encryption key.

**Step 5:** Deriving the encryption key, the final encryption key is obtained by iteratively applying an XOR operation with the static and chaotic primary keys. Figure 8 shows the resulting encryption key.

**Figure 9:**
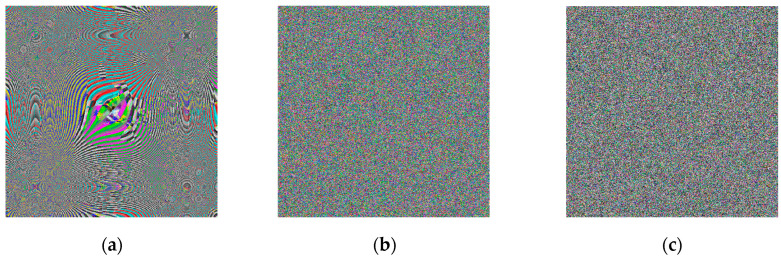
Encrypted images: (**a**) encoded image at the first stage; (**b**) encoded image after the scrambling process; (**c**) shows the final encoded image.

**Figure 10:**
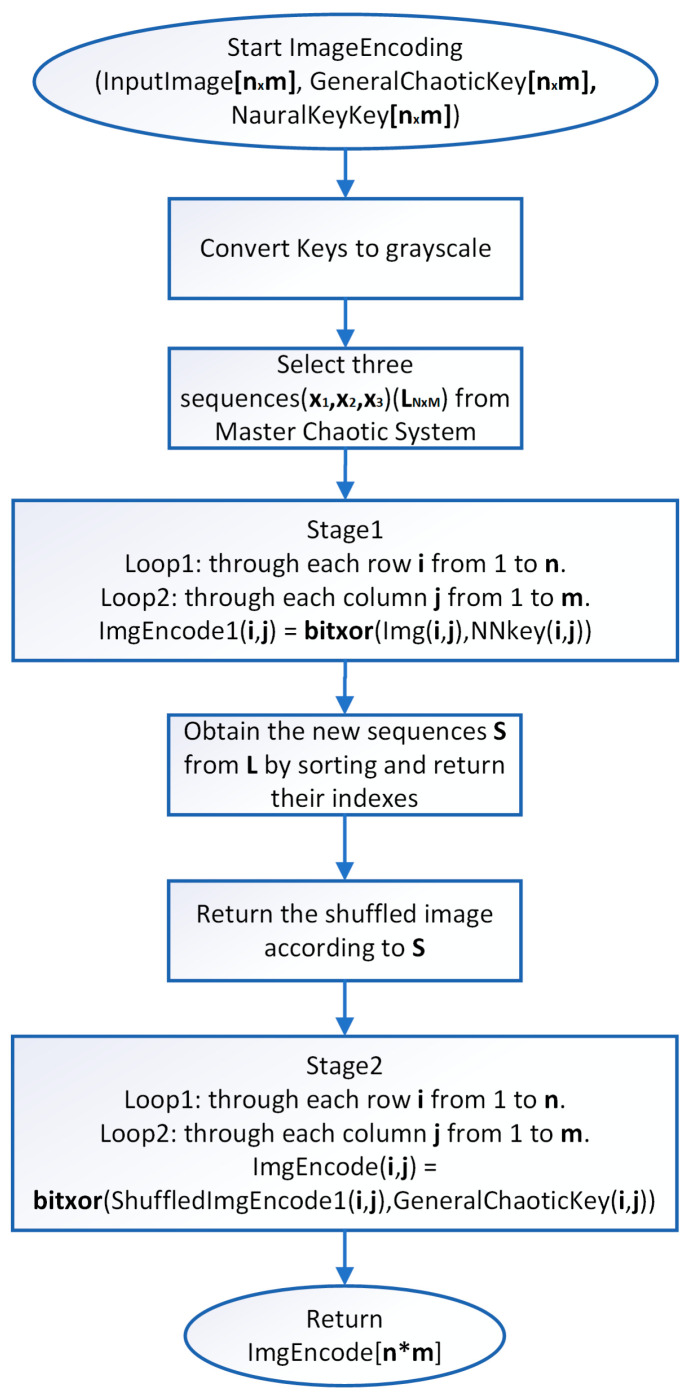
Block diagram of generalized image encoding.

**Step 6:** Image encoding—As with most encoding methods, here, the encoding key (image) is paired with the input image using an XOR operation, thus obtaining a kind of masking of the image. Here, the process is carried out in two stages—initially the image is paired with the neural key, and then with the general chaotic key. Between the two steps, the image already encoded with the neural key is subjected to a shuffling process. Shuffling begins with dividing the image into the three-color channels. Each of the new images obtained is shuffled by using the chaotic signal to obtain a numerical sequence with a length equal to the size of the image. The indices of this sequence are used for shuffling. The three images thus obtained are combined again for pairing with the chaotic key. Figure 9a shows the encoded image at the first stage, Figure 9b shows the encoded image after the scrambling process, and Figure 9c shows the final encoded image. A simple block diagram for the final process of image encoding via the general chaotic encryption key is presented in Figure 10.

**Figure 11:**
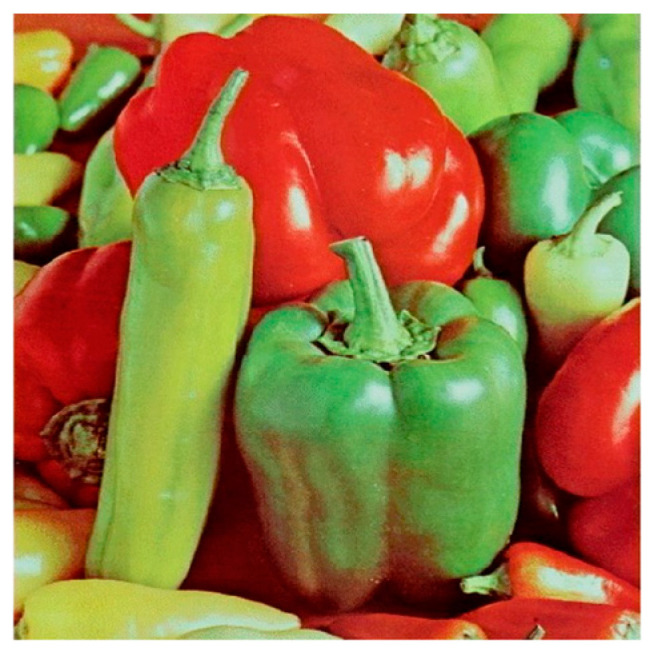
Decrypted image.

**Step 7:** Unlike traditional OTP methods, here, the process of chaotic synchronization is of primary importance and is executed first. Through the functional dependencies described above, the decoding static, chaotic, and neural keys are obtained, followed by the final main decoding key. The decryption procedure is carried out by generating the same chaotic sequence used during encoding, through the synchronized chaotic generator. A reverse index array of the chaotic sequence is formed to restore the original arrangement of the pixels across the three-color channels. Inverse shuffling is applied based on these indices. The actual decoding is again performed using an XOR operation, meaning that at its core, the technique represents a one-to-one encryption-decryption algorithm, and no loss of information or quality of the decoded image is expected. The presented scheme can operate either with key exchange or in a hybrid mode, where only part of the information required for generating the decoding keys (for the static and neural keys) is shared. Figure 11 shows the resulting decoded image.

**Figure 12:**
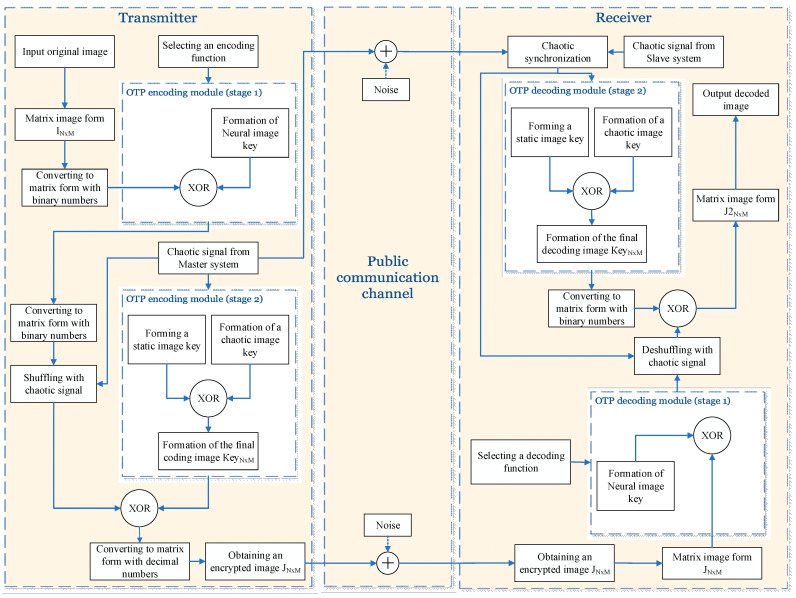
Generalized block diagram of a modified OTP (One-time path) algorithm for image encryption with an implemented chaotic synchronization scheme and ANNs.

A generalized block diagram is shown in Figure 12.

## 4. Examples, Results, and Analysis

In this section, an application of the proposed coding scheme will be presented by implementing a synchronization scheme between two identical Chua systems and training an ANN to approximate three randomly selected coding functions. Results from testing the proposed coding scheme and subsequent analyses based on the main criteria for assessing the quality of image coding are presented.

### 4.1. Synchronization Between Two Identical Chaotic Chua Systems

#### 4.1.1. Description of the Chaotic Model

**Figure 13:**
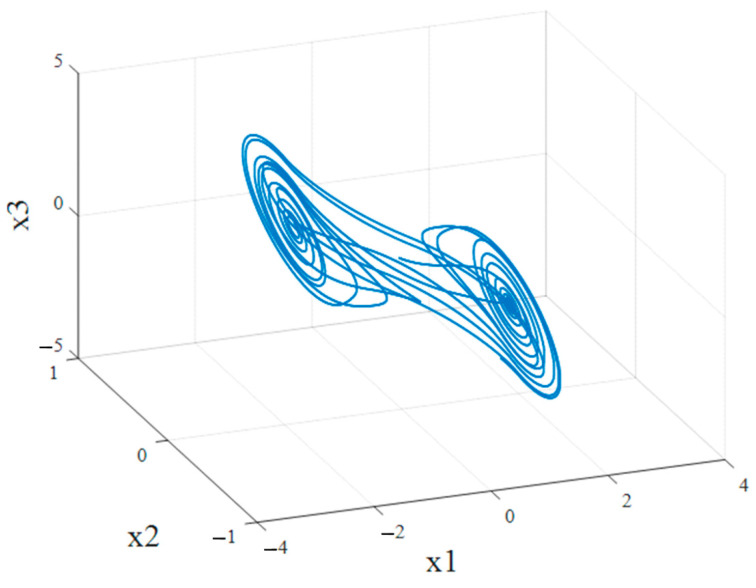
Chaotic attractor of Chua’s system.

The Chua system [48] is a model of a nonlinear electrical circuit. It is the most exploited chaotic system in practical applications because of its simplicity, easy circuit implementation, and at the same time the system clearly exhibits all the fundamental characteristics of chaotic behavior. The equations of the basic Chua system are

(23) $$\left\{\begin{array}{c} {\dot{x}}_{1}=\alpha [x_{2}-x_{1}-f(x_{1})]\\ {\dot{x}}_{2}=x_{1}-x_{2}+x_{3}\;\;\;\;\;\;\;\;\;\;\;\\ {\dot{x}}_{3}=-\beta x_{2}\;\;\;\;\;\;\;\;\;\;\;\;\;\;\;\;\;\;\;\;\;\;\;\; \end{array}\right.\;$$

where the optimal parameter values for the system are α=10,β=14.87. Nonlinearity is $f(x_{1})=bx_{1}+\frac{a-b}{2}\left(\left|x_{1}+1\right|-\left|x_{1}-1\right|\right)$, a=−1.27, b=−0.68. Figure 10 shows the attractor under the following arbitrarily chosen initial conditions: $x_{0}={\left[\begin{array}{ccc} 0.1 & 0.1 & 0 \end{array}\right]}^{T}$. Observing its dynamics in the state space, it is seen that it has a chaotic attractor (Figure 13), i.e., chaotic oscillations arise in the system.

#### 4.1.2. Transformation and Synchronization

The subordinate system has the following form:

(24) $$\left\{\begin{array}{c} {\dot{\tilde{x}}}_{1}=\alpha \left[{\tilde{x}}_{2}-{\tilde{x}}_{1}-f\left({\tilde{x}}_{1}\right)\right]+u\\ {\dot{\tilde{x}}}_{2}={\tilde{x}}_{1}-{\tilde{x}}_{2}+{\tilde{x}}_{3}\;\;\;\;\;\;\;\;\;\;\;\;\;\;\;\;\;\;\;\;\\ {\dot{\tilde{x}}}_{3}=-\beta {\tilde{x}}_{2}\;\;\;\;\;\;\;\;\;\;\;\;\;\;\;\;\;\;\;\;\;\;\;\;\;\;\;\;\;\;\;\;\;\; \end{array}\right.$$

When transforming the Slave system, the result is

(25) $$L_{g}L_{f}^{0}h\left(\tilde{x}\right)=\frac{\partial h\left(\tilde{x}\right)}{\partial \tilde{x}}.g(\tilde{x})=0$$

(26) $$L_{g}L_{f}^{1}h\left(\tilde{x}\right)=L_{g}\left(\frac{\partial h\left(\tilde{x}\right)}{\partial \tilde{x}}.f(\tilde{x})\right)=0$$

(27) $$L_{g}L_{f}^{2}h\left(\tilde{x}\right)=L_{g}L_{f}\left(L_{f}h\left(\tilde{x}\right)\right)=\left(\frac{\partial L_{f}^{1}h\left(\tilde{x}\right)}{\partial \tilde{x}}.f\left(\tilde{x}\right)\right)g\left(\tilde{x}\right)=-\beta$$

Thus for the transformation map, taking into account (25)–(27) we obtain

(28) $$S_{1}\left(\tilde{x}\right)=h\left(\tilde{x}\right)={\tilde{x}}_{3}=z_{1}\;\;\;\;\;\;\;\;\;\;\;\;\;\;\;\;\;\;\;\;\;\;\;\;\;\;\;\;\;\;\;\;\;\;S_{2}\left(\tilde{x}\right)=L_{f}h\left(\tilde{x}\right)=-\beta {\tilde{x}}_{2}=z_{2}\;\;\;\;\;\;\;\;\;\;\;\;\;\;\;\;\;\;\;\;\;\;\;\;S_{3}\left(\tilde{x}\right)=L_{f}^{2}h\left(\tilde{x}\right)=-\beta \left({\tilde{x}}_{1}-{\tilde{x}}_{2}+{\tilde{x}}_{3}\right)=z_{3}$$

After brief mathematical transformations, we obtain

(29) $$a\left(\tilde{z}\right)=L_{g}L_{f}^{2}h\left(\tilde{x}\right)=-\beta$$

(30) $$b\left(\tilde{z}\right)=L_{f}^{3}h\left(\tilde{x}\right)=-\beta \alpha {\tilde{x}}_{2}+\beta \alpha {\tilde{x}}_{1}+\beta \alpha f\left({\tilde{x}}_{1}\right)+\beta {\tilde{x}}_{1}-\beta {\tilde{x}}_{2}+\beta {\tilde{x}}_{3}+\beta^{2}{\tilde{x}}_{2}$$

(31) $$u=\frac{1}{-\beta }\left(\beta \alpha {\tilde{x}}_{2}-\beta \alpha {\tilde{x}}_{1}-\beta \alpha f\left({\tilde{x}}_{1}\right)-\beta {\tilde{x}}_{1}+\beta {\tilde{x}}_{2}-\beta {\tilde{x}}_{3}-\beta^{2}{\tilde{x}}_{2}+v\right)$$

The control system is transformed in a similar way:

(32) $$L_{g}L_{f}^{0}h\left(x\right)=\frac{\partial h\left(x\right)}{\partial x}.g(x)=0$$

(33) $$L_{g}L_{f}^{1}h\left(x\right)=L_{g}\left(\frac{\partial h\left(x\right)}{\partial x}.f(x)\right)=0$$

(34) $$L_{g}L_{f}^{2}h\left(x\right)=L_{g}L_{f}\left(L_{f}h\left(x\right)\right)=\left(\frac{\partial L_{f}^{1}h\left(x\right)}{\partial x}.f\left(x\right)\right)g\left(x\right)=-\beta$$

With the following transformation map:

(35) $$S_{1}\left(x\right)=h\left(x\right)=x_{3}=z_{1}\;\;\;\;\;\;\;\;\;\;\;\;\;\;\;\;\;\;\;\;\;\;\;\;\;\;\;\;\;\;\;\;\;\;S_{2}\left(x\right)=L_{f}h\left(x\right)=-\beta x_{2}=z_{2}\;\;\;\;\;\;\;\;\;\;\;\;\;\;\;\;\;\;\;\;\;\;\;\;S_{3}\left(x\right)=L_{f}^{2}h\left(x\right)=-\beta \left(x_{1}-x_{2}+x_{3}\right)=z_{3}$$

and

(36) $$b\left(z\right)=L_{f}^{3}h\left(x\right)=-\beta \alpha x_{2}+\beta \alpha x_{1}+\beta \alpha f\left(x_{1}\right)+\beta x_{1}-\beta x_{2}+\beta x_{3}+\beta^{2}x_{2}.$$

An error vector is defined $e=\tilde{z}-z$, respectively $\dot{e}=\dot{\tilde{z}}-\dot{z},$ where for the system of disagreement we obtain

(37) $$\left\{\begin{array}{c} {\dot{e}}_{1}=C_{1}\left({\dot{\tilde{z}}}_{1}-{\dot{z}}_{1}\right)=C_{1}e_{1}\;\;\;\;\;\;\;\;\;\;\;\;\;\;\;\;\;\;\;\;\;\;\;\;\;\;\;\;\;\;\;\;\;\;\;\;\;\;\;\;\;\;\;\;\;\;\;\;\;\;\;\;\;\;\;\;\;\;\;\;\;\;\;\;\;\;\;\;\;\;\;\;\;\;\;\;\;\;\;\;\;\;\;\;\;\;\;\;\;\;\;\;\;\;\;\;\;\;\;\;\;\;\;\;\;\\ {\dot{e}}_{2}=C_{2}({\dot{\tilde{z}}}_{2}-{\dot{z}}_{2})=C_{2}e_{2}\;\;\;\;\;\;\;\;\;\;\;\;\;\;\;\;\;\;\;\;\;\;\;\;\;\;\;\;\;\;\;\;\;\;\;\;\;\;\;\;\;\;\;\;\;\;\;\;\;\;\;\;\;\;\;\;\;\;\;\;\;\;\;\;\;\;\;\;\;\;\;\;\;\;\;\;\;\;\;\;\;\;\;\;\;\;\;\;\;\;\;\;\;\;\;\;\;\;\;\;\;\;\;\;\;\\ {\dot{e}}_{3}=C_{3}e_{3}-\beta \alpha e_{2}+\beta \alpha e_{1}+\beta \alpha f\left({\tilde{x}}_{1}\right)+\beta e_{1}-\beta e_{2}+\beta e_{3}+\beta^{2}e_{2}-\beta -\beta \alpha f\left(x_{1}\right) \end{array}\right.$$

The coefficients $C_{1÷3}$ are coefficients of the Hurwitz polynomial and are chosen as follows: $C={\left[\begin{array}{ccc} 30 & 30 & 10 \end{array}\right]}^{T}$. With the following synthesized control, the system becomes asymptotically stable:

(38) $$u=\frac{1}{-\beta }\left(-\beta \alpha e_{2}+\beta \alpha e_{1}+\beta \alpha f\left({\tilde{x}}_{1}\right)+\beta e_{1}-\beta e_{2}-\beta e_{3}+\beta^{2}e_{2}-\beta \alpha f\left(x_{1}\right)+Ce\right)$$

**Figure 14:**
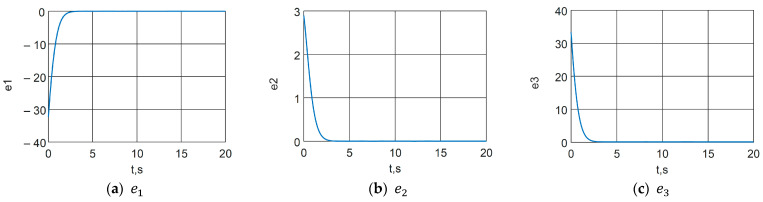
Error functions of the synchronization scheme.

**Figure 15:**
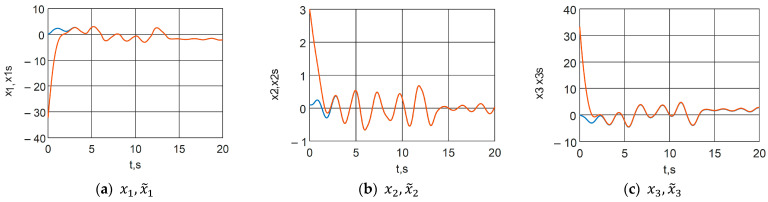
Time functions of the Master (blue line) and Slave (red line) chaotic systems.

A synchronization scheme with a control system (23), a slave system (24) and a control (38) is implemented. The following arbitrary initial conditions are chosen: $x_{0}={\left[\begin{array}{ccc} 0.1 & 0.1 & 0 \end{array}\right]}^{T}$ and ${\tilde{x}}_{0}={\left[\begin{array}{ccc} 1 & 3 & 0 \end{array}\right]}^{T}$. The error functions are graphically presented in Figure 14, where a transient process of approximately 3 s is observed. From Figure 15, which presents the time characteristics of the Master and Slave systems, the occurrence of identical synchronization can be graphically confirmed.

### 4.2. Training ANNs and Approximating Temporal Coding Functions

In this section, we present the simulation results of the training of the selected ANN and the subsequent approximation of different functions with one variable. The used ANN, represented by the architecture in Figure 2, is trained to reproduce three functions of increasing complexity, which are, respectively,

(39) $$\begin{array}{c} f_{1}\left(x\right)=4.3x^{2}+1\;\;\;\;\;\;\;\;\;\;\;\;\;\;\;\;\;\;\;\;\;\;\;\;\;\;\;\;\;\;\;\;\;\;\;\;\;\;\;\;\;\;\;\;\;\;\;\;\;\\ f_{2}\left(x\right)=3x^{3}+0.1x^{2}+x+1\;\;\;\;\;\;\;\;\;\;\;\;\;\;\;\;\;\;\;\;\;\;\;\;\;\;\;\;\;\\ f_{3}\left(x\right)=\frac{3x^{4}+2x^{3}+2.1x^{2}+5x+3\sin\left(x^{3}\right)}{2x.e^{x}} \end{array}$$

**Figure 16:**
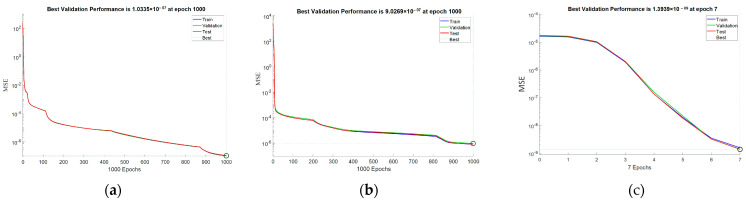
Errors from training the ANN with the three coding functions: (**a**) For function *f*_1_(t); (**b**) For function *f*_2_(t); (**c**) For function *f*_3_(t).

According to the limits of variation of Xm1, which depends on the limits of variation of initial conditions of the systems of the controlling and subordinate chaotic system, the ANN is chosen to reproduce with the desired accuracy *f*_1_(t), *f*_2_(t) and *f*_3_(t) in the range from −3 to 3, with a variation step of 0.001. This provides a sufficiently large number of values allowing to encode very large images (for example, with a maximum size of 6000 × 6000), since the encoding image is formed by generating coordinates along x by y using a linear spatial vector with values from the approximated function itself. The change of the chaotic system itself does not require a change in the selected encoding function, but changing the range of variation of the function with which to work is an additional element that can be used to increase the degree of complexity of the encoding. As training parameters, it is chosen to conduct training with a desired error of at least 0.001 (goal = 1 × 10^−3^), with a maximum set of 1000 epochs (epochs = 1000). In order to evaluate the results of the approximation, an error criterion is required. The normalized MRS error function, which is calculated during training from the validation data, is chosen as the error criterion. Usually, in the training process, a percentage ratio is chosen of what part of the data is used for training (those that are fed to the ANN during training), validation (used to measure the state to stop training) and testing (used for independent testing of the ANN during and after training, and are not related to the training process). The percentage ratio used is 70% for training, 15% for validation and 15% for testing. Figure 16 shows the graphs of the ANN training errors for the three coding functions.

**Figure 17:**
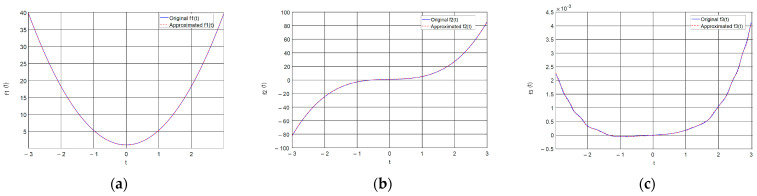
Approximation of sample time functions: (**a**) Function *f*_1_(t); (**b**) Function *f*_2_(t); (**c**) Function *f*_3_(t).

**Figure 18:**
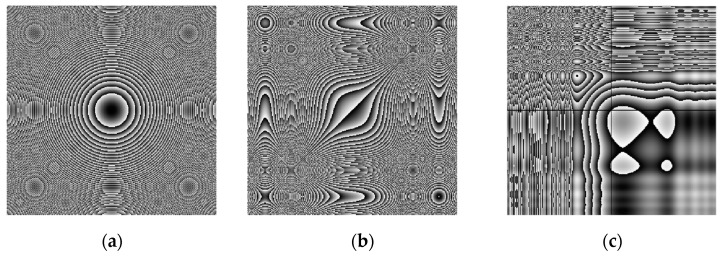
Formed coding images: (**a**) by function *f*_1_(t); (**b**) by function *f*_2_(t); (**c**) by function *f*_3_(t).

Figure 17 and Figure 18 present a comparison of the original with the approximated function and the subsequent formed coding images, respectively. Numerical results from the training process of ANNs are shown in Table 1. Figure 17 clearly shows that the ANN approximates the selected functions in a very sufficient manner. This is also proven by Figure 16, which shows that the average MSE is of the order of 1 × 10^-7^ or lower, which is sufficient for current purposes. The selected coding functions allow the formation of good coding images, the effectiveness of which will be investigated in the next section.

### 4.3. Examples and Results

The main results achieved with the three encoding functions for the same test image (“Peppers”) are presented in Section 4.3.1. They include both graphical interpretations—histograms and correlations, and numerical results—information entropy, correlation coefficient, UACI and NPCR. Section 4.3.2 presents the results achieved with other test images from referenced open-access databases. Studies have been conducted regarding the robustness of the proposed modified image encoding algorithms against various cryptographic attacks (Section 4.4.3). Section 4.4.4 provides an analysis and summary of the obtained graphical and numerical results.

#### 4.3.1. Main Results

The main graphical and numerical results achieved with the developed modified algorithm are presented for each of the coding functions.

- Results obtained with encrypting function: $f_{1}\left(x\right)=4.3x^{2}+1$.
- Results obtained with encrypting function: $f_{2}\left(x\right)=3x^{3}+0.1x^{2}+x+1$.
- Results obtained with encrypting function: $f_{3}\left(x\right)=\frac{3x^{4}+2x^{3}+2.1x^{2}+5x+3\sin\left(x^{3}\right)}{2x.e^{x}}$.

**Figure 19:**
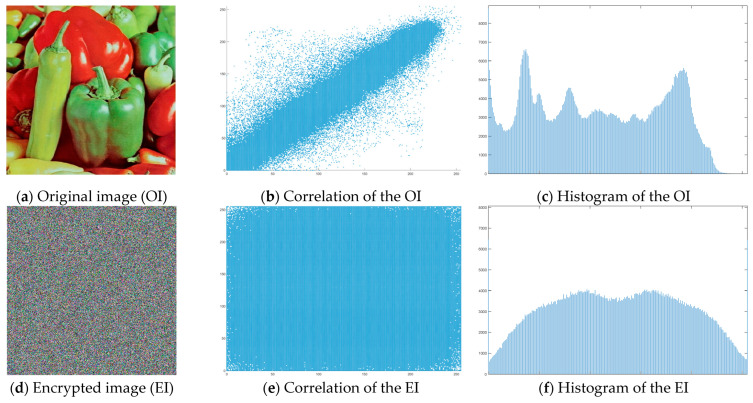
Histograms and correlations for original and encrypted images obtained with encrypting function $f_{1}\left(x\right)$.

**Figure 20:**
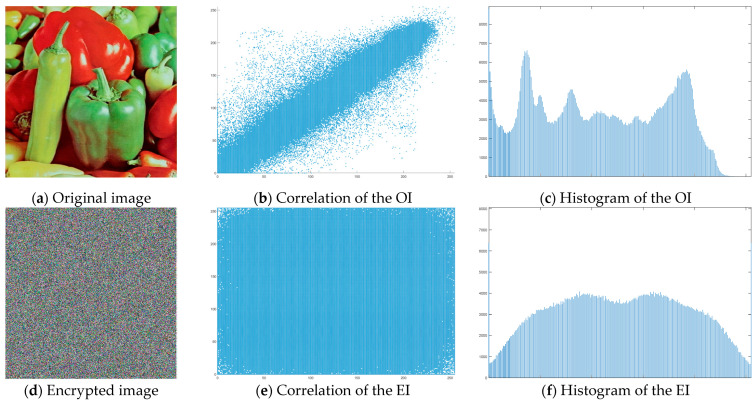
Histograms and correlations for original and encrypted images obtained with encrypting function $f_{2}\left(x\right)$.

**Figure 21:**
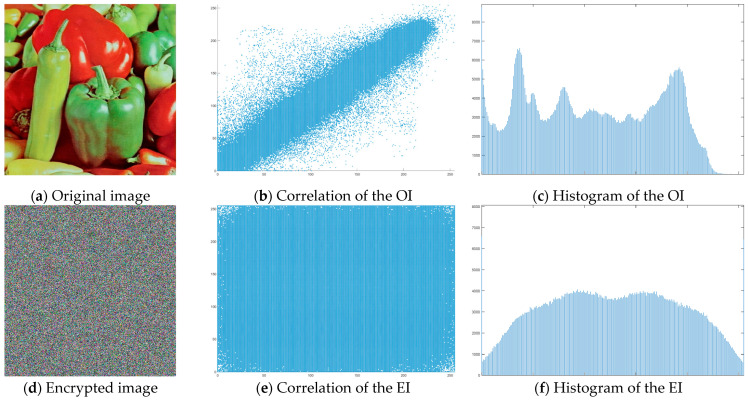
Histograms and correlations for original and encrypted images obtained with encrypting function $f_{3}\left(x\right)$.

The “Peppers” image was used as the main test image, as an established standard for testing encryption algorithms. Graphical representation of histograms and correlations between original and encrypted image are shown in Figure 19—$f_{1}$, Figure 20—$f_{2}$ and Figure 21—$f_{3}$. From the presented graphical results, it is clearly observed that there is no region of attraction when correlating the encrypted image with the applied synchronization scheme for all three coding functions. This is also proven numerically as the average correlation value of the encrypted image is −0.0014.

The second part of the graphical results, based on the histograms of the original and encrypted images, also confirms the high degree of protection, since a relatively smooth distribution of the pixel brightness value is observed in the encrypted image.

The numerical results for the information entropy and the coefficients NPCR and UACI (presented in Table 2—$f_{1}$, Table 3—$f_{2}$ and Table 4—$f_{3}$)—are close to the theoretically maximum values and confirm the qualities of the modified algorithm.

**Figure 22:**
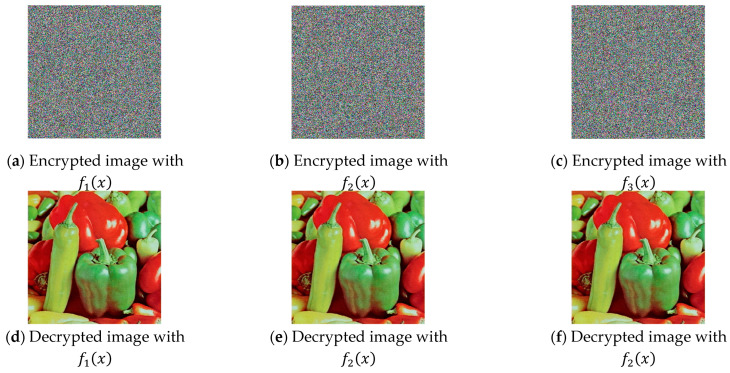
Encrypted and decrypted pepper images.

Figure 22 shows the decrypted images using the three coding functions. It is clearly observed that the image is restored without loss of quality and information, which is expected given the type of transients obtained when implementing chaotic synchronization.

#### 4.3.2. Additional Results

When testing the encryption algorithm developed above, images that are common in scientific publications on the current topic are used. These images are found in pre-selected databases of test images. Test images are in many cases chosen to represent natural or typical images that certain image coding techniques have to deal with. These images meet certain requirements with which the quality indicators of the used coding algorithms are examined. Images with a single main object on a heterogeneous background, images with multiple objects and a homogeneous background, as well as monochromatic (black and white) images are used. For the most part, these images are characterized by variable image brightness with an inverse character, incomplete dynamic range and the presence of blur and noise of a heterogeneous type. For this reason, they pose a number of challenges to image processing algorithms, such as reproducing fine details and textures, sharp transitions and edges, and unifying areas.

**Figure 23:**
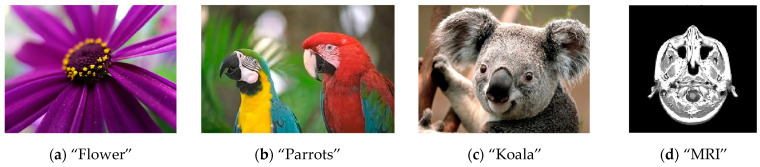
Test images.

In this paper, some of the most commonly used test images have been selected (Figure 23), namely,

Following are table presentations of original and encrypted images with their graphical results for histograms (Table 5) and correlations (Table 6), numerical results for information entropy (Table 7), and correlation (Table 8).

### 4.4. Security Analysis

#### 4.4.1. Brute-Force Attack

To analyze the resistance of the coding algorithm to a brute force attack, the size of the keyspace is calculated [49]. It is believed that for sufficient stability, the algorithm should have a keyspace > 2^100^. The proposed modification of the algorithm uses a chaotic Chua system with four parameters (α, β, a, b) and initial conditions N_0_, taking into account the accuracy standard of 64-bit floating-point numbers, which is 10^15^, then to obtain the chaotic key keyspace = N_0_ × 10^15×4^.

Obtaining the neural key is implemented by approximating functions, if we take the average in size, which is a 4th order polynomial and has 4 coefficients, then, here, keyspace = N_0_ × 10^15×4^, and if we consider the generated image of a code mask, with a minimum size of a meaningful image of 16 × 16, then keyspace = 2^8×16×16^, but since the algorithm has practically no limit on the coding functions used, it is appropriate to consider the number of coefficients.

The use of the static key is a basic element of OTP algorithms, which is taken as a coding image and keyspace = 2^8×16×16^.

Thus, the total number of theoretically possible encryption keys will be greater than (N_0_ × 10^15×4^) × (10^15×4^) × (2^8×16×16^) = 10^60^ × 10^60^ × 2^2048^ = 2^398+2048^ = 2^2446^ or 10^736^, which meets the criterion for resistance to Brute-Force attack.

#### 4.4.2. Differential Attack

Differential Cryptanalysis is a cryptanalysis method used to break symmetric encryption algorithms, especially those based on block ciphers. It is based on analyzing the differences in the output data when small changes are applied to the initial input, providing the ability to trace the dependencies between the differences in the input and output values in order to reveal weaknesses in the algorithm.

The coefficients needed to perform the analysis are

- NPCR (Number of Pixels Change Rate)—measures the percentage of pixels that have changed their value in the encrypted image when the original image has undergone minimal modification. A high NPCR value (close to 99%) indicates that the encryption algorithm is resistant to small changes in the original image;
- UACI (Unified Average Changing Intensity)—measures the average change in pixel intensity between the original encrypted image and its modified version, assessing the impact of a small change in the original image (for example, changing the value of one pixel). A high UACI value indicates that the encryption algorithm is sensitive to small changes in the original image. A typical value for effective encryption algorithms is in the range of 20—33%.

These coefficients represent a kind of assessment of encryption algorithms, as they indicate whether the algorithm has the necessary sensitivity to changes in the data. High values of NPCR and UACI demonstrate that small changes in the original image lead to significant differences in the encrypted image, making attacks more difficult. The NPCR and UACI coefficients are also used for comparative analysis between different encryption methods.

Table 9 contains comparisons of NPCR, UACI, correlation and entropy across the three encoding functions.

#### 4.4.3. Noise and Data Cut Attacks

When transmitting encrypted images over unprotected communication channels, ensuring robustness against noise and data clipping attacks is a top priority. This experiment was conducted to test the robustness of the proposed modified algorithm against noise and data clipping attacks. In the course of the experiment, an image encrypted using the proposed algorithm was contaminated with “salt and pepper” noise at two different levels—5% and 10%, and then decrypted. The encrypted images were also subjected to a clipping attack with sizes 57 × 57 and 170 × 170 (equivalent to 10% and 25% loss of the encoded image) and then decrypted. The obtained results are presented in Table 10 and Table 11.

To measure the difference between the obtained and the original image, the values of MSE and PSNR can be used. MSE (Mean Squared Error) is a measure of the average difference between the corresponding pixels of two images. The higher the MSE value, the greater the difference between the two images. PSNR measures the quality of the image by comparing the maximum possible pixel value with the level of error (noise). A high PSNR value results in less distortion and higher image quality. In cryptographic applications, the goal is to keep the PSNR low, so the encrypted image differs significantly from the original.

#### 4.4.4. Key Sensitivity

Reliable image encryption algorithms should demonstrate high sensitivity to secret keys, which leads to a noticeable change in the decrypted image with minimal changes in the initial conditions of the secret key used during the encryption process. This study was conducted both with a change in the initial conditions and with a change in the system parameters. The encryption algorithm was run with initial conditions $x_{0}={\left[\begin{array}{ccc} 0.1 & 0.1 & 0 \end{array}\right]}^{T}$ and system parameters α=10,β=14.87, a=−1.27 и b=−0.68. Figure 21a shows the original image, the encrypted image is shown in Figure 21b, and the resulting decrypted image is shown in Figure 21c. A decrypted image after a change in the initial conditions $x_{0}={\left[\begin{array}{ccc} 0.1 & 0.1 & 0.0000001 \end{array}\right]}^{T}$ is presented in Figure 21d, and Figure 21e presents a decrypted image after a change in the system parameters α=10.0000001,β=14.87, a=−1.27 и b=−0.68.

**Figure 24:**
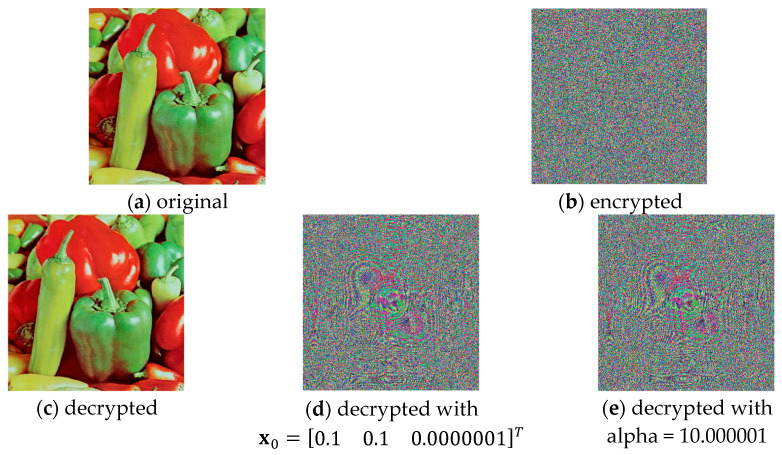
Encrypted and decrypted images.

From Figure 24, it is clear that the proposed algorithm is extremely sensitive even to minimal changes, which is to some extent due to the use of a chaotic system and proves a high level of key security.

#### 4.4.5. Computational Complexity

When estimating the complexity of an encryption algorithm, the notation O(…) is usually used. This notation “simplifies” the algorithm by leaving only the most significant characteristic, i.e., the function on which the execution time depends the most, in other words, on which it grows the fastest when the size of the input data increases.

The process of encoding an image with dimension N*M is divided into several parts that are of fundamental importance:

- Opening and obtaining an image—Computational Complexity O(N*M).
- Static key generation—The process is iterative with a maximum number of iterations N*M*8 and it follows that the computational complexity is O(N*M*8).
- Chaotic key generation—N*M values are taken from the chaotic signal, i.e., the complexity is O(N*M).
- Generating a neural key—In neural networks with a function approximation task, the number of hidden layers and neurons is less than the number of inputs T and the complexity is determined by the number of inputs—O(T) for T << N*M. But at this stage, the image is encoded with the resulting neural key, which is also an image of size N*M, so the complexity is O(N*M).
- Obtaining an encoding key and overall encoding—the operation used is BitXOR, i.e., complexity O(N*M).

From the operations thus distinguished, it can be said that the entire time complexity of the algorithm is O(N*M*8).

### 4.5. Analysis of the Obtained Results

The analysis of the obtained data is based on the calculation of the coefficients described above in order to assess the achieved results from the application of the modified image encryption algorithm.

In terms of information entropy, the value for the encrypted images is close to the theoretical maximum—8, which means a high degree of randomness and is a desired result for cryptographic algorithms. The largest absolute difference is obtained for the MRI image—approximately 4.3, which is due to the homogeneity of this type of image. Similar types of images with low initial entropy show a significant increase after encryption.

Table 8 shows the correlations for original and encrypted images. Their graphical interpretation is presented in Table 6. It can be summarized that the original images have a high correlation of the order of 0.91–0.98, which is an expected value for natural images, where neighboring pixels have similar values. When encrypting images, the correlation coefficient is close to zero and even negative, which indicates an effective violation of the connection between neighboring pixels and testifies to the high level of protection. Obtaining similar values indicates a successful violation of the correlation between pixels, making the encrypted images resistant to statistical attacks.

The values of NPCR and UACI presented in Table 8 are extremely high—NPCR≈99.60÷99.74 и $\bar{UACI}\approx 33.54$. These values demonstrate significant changes in the encrypted images and are an indicator of high security.

Applying different encryption functions gives similar results in practice. The possibility of using different functions, giving good encoding results, makes the scheme extremely applicable since there is practically no restriction in the choice of the encoding function and its parameters. The same applies to the choice of chaotic systems to be synchronized. This is a positive aspect for any encoding algorithm.

A security analysis was performed based on many different cryptographic attacks. The obtained results indicate a very high level of encryption, which can be confirmed by comparing the proposed modified algorithm with other encryption algorithms: Hosny et al. [50], Chen et al. [51], Benaissi et al. [49], Gao et al. [52], Kumar et al. [53]. The “Peppers” test image (512 × 512) was used for the comparison. The results are presented in Table 11.

From Table 12, it can be concluded that the presented encryption scheme is not inferior in quality to other similar algorithms presented in reputable scientific publications, and in some indicators it even performs better.

## 5. Conclusions

This article considers the problem of image protection. A modified image encryption scheme is that combines the classical OTP algorithm, the advantages of chaotic synchronization schemes, and the integration of ANNs is presented. The proposed approach differs from the traditional one by implementing multilayering provided by the formation and combination of a static, chaotic, and neural key, which is an innovative approach in the field of image encryption. This multilayer approach is achieved by implementing ANNs to conceal the original source of obtaining the encoding key, in the context of the classical function approximation task.

The results obtained and the tests and analyses performed are based on generalizations of numerical and graphical data for histograms, information entropy (7.9975 ÷ 7.9994), NPCR (99.6200 ÷ 99.8631), UACI (32.8354 ÷ 34.9938), and correlation coefficients (−0.0057 ÷ 0.0012), as well as an analysis of the resistance to various cryptographic attacks. The results obtained prove they achieved a high level of protection tending to the theoretical maximum.

The proposed modified image encryption scheme successfully integrates innovative approaches and classical methods, achieving high stability and flexibility. The flexibility of the proposed approach lies in the choice of chaotic systems, coding functions, neural network architectures, etc., which provide the possibility of customizing the algorithm to the specific requirements of the application. The testing results confirm the successful implementation of the approach for the secure transmission of sensitive information in real applications.

## Figures and Tables

**Table 1:** Numerical results from the ANN training process.

Function	Epochs	MSE	Data Split
$f_{1}$	1000	1.0335 × 10^−7^	70/15/15
$f_{2}$	1000	9.0269 × 10^−7^	70/15/15
$f_{3}$	7	1.3939 × 10^−9^	70/15/15

**Table 2:** Numerical analysis of the encrypted image with encryption function: $f_{1}\left(x\right)$.

Entropy		Correlation		Metric Indicators	
Original	Encrypted	Original	Encrypted	NPCR	UACI
7.7356	7.9992	0.0765	−0.0014	99.7339	33.2500

**Table 3:** Numerical analysis of the encrypted image with encryption function: $f_{2}\left(x\right)$.

Entropy		Correlation		Metric Indicators	
Original	Encrypted	Original	Encrypted	NPCR	UACI
7.7356	7.9993	0.0765	−0.0002	99.8631	33.5565

**Table 4:** Numerical analysis of the encrypted image with encryption function: $f_{3}\left(x\right)$.

Entropy		Correlation		Metric Indicators	
Original	Encrypted	Original	Encrypted	NPCR	UACI
7.7356	7.9994	0.0765	0.0001	99.7252	33.3607

**Table 5:** Histograms of original and encrypted images.

		Function 1		Function 2		Function 3	
Original Image	Histogram of the OI	Encrypted Image	Histogram of the EI	Encrypted Image	Histogram of the EI	Encrypted Image	Histogram of the EI
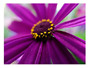	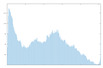		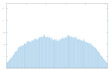	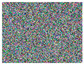	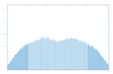	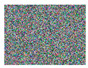	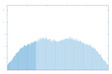
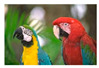	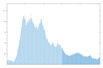	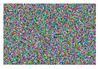	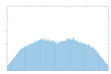	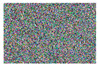	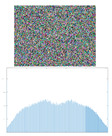	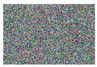	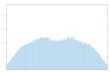
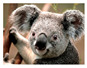	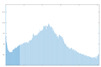	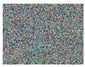	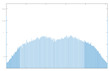	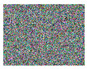	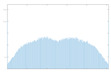	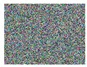	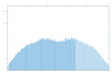
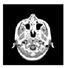	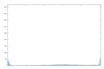	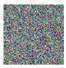	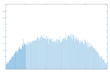	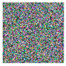	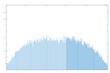	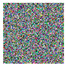	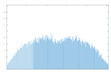

**Table 6:** Correlations of original and encrypted images.

		Function 1		Function 2		Function 3	
Original Image	Corelation of the OI	Encrypted Image	Corelation of the EI	Encrypted Image	Corelation of the EI	Encrypted Image	Corelation of the EI
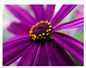	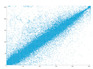	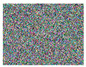	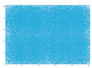	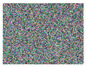	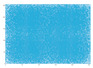	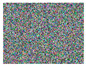	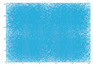
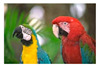	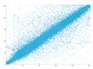	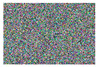	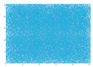	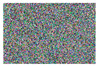	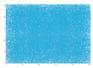	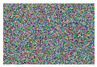	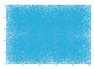
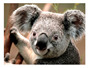	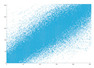	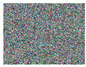	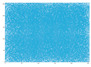	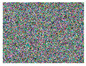	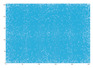	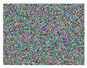	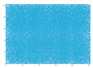
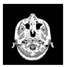	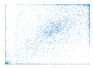	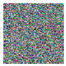	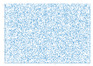	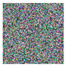	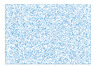	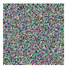	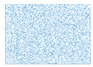

**Table 7:** Numerical results for the informational entropy of original and encrypted images.

Function	Image	Original	Encrypted	Decrypted
$f_{1}\left(x\right)$	flower	7.3961	7.9978	7.3961
	parrot	7.4122	7.9979	7.4122
	koala	7.8018	7.9980	7.8018
	mri	3.6779	7.9881	3.6779
$f_{2}\left(x\right)$	flower	7.3961	7.9975	7.3961
	parrot	7.4122	7.9977	7.4122
	koala	7.8018	7.9980	7.8018
	mri	3.6779	7.9889	3.6779
$f_{3}\left(x\right)$	flower	7.3961	7.9980	7.3961
	parrot	7.4122	7.9978	7.4122
	koala	7.8018	7.9980	7.8018
	mri	3.6779	7.9884	3.6779

**Table 8:** Correlation analysis of original and encrypted images.

Function	Image	Original	Encrypted	Decrypted
$f_{1}\left(x\right)$	flower	0.9798	0.0006	0.9798
	parrot	0.9735	0.0003	0.9735
	koala	0.9114	0.0003	0.9114
	mri	0.9365	−0.0014	0.9365
$f_{2}\left(x\right)$	flower	0.9798	−0.0022	0.9798
	parrot	0.9735	−0.0057	0.9735
	koala	0.9114	−0.0018	0.9114
	mri	0.9365	0.0100	0.9365
$f_{3}\left(x\right)$	flower	0.9798	0.0012	0.9798
	parrot	0.9735	−0.0005	0.9735
	koala	0.9114	0.0011	0.9114
	mri	0.9365	−0.0032	0.9365

**Table 9:** Numerical analysis of encrypted images.

Function	Image	Entropy	Correlation	NPCR	UACI
$f_{1}\left(x\right)$	peppers	7.9992	−0.0014	99.7339	33.2500
	flower	7.9978	0.0006	99.6540	33.6301
	parrot	7.9979	0.0003	99.6253	33.5995
	koala	7.9980	0.0003	99.6200	32.8963
	mri	7.9881	−0.0014	99.7488	33.3001
$f_{2}\left(x\right)$	peppers	7.9993	−0.0002	99.8631	33.5565
	flower	7.9975	−0.0022	99.6290	33.4312
	parrot	7.9977	−0.0057	99.6263	33.7735
	koala	7.9980	−0.0018	99.6245	32.8354
	mri	7.9889	0.0010	99.6460	34.9938
$f_{3}\left(x\right)$	peppers	7.9994	0.0001	99.7252	33. 3607
	flower	7.9980	0.0012	99.6200	33.5966
	parrot	7.9978	−0.0005	99.6305	33.7961
	koala	7.9980	0.0011	99.6256	32.8455
	mri	7.9884	−0.0032	99.6094	33.8988

**Table 10:** Results from “Salt and pepper” noise analysis.

“Salt and Pepper” Noise	Original	Encrypted	Decrypted	PSNR
5%	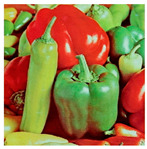	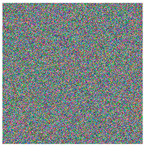	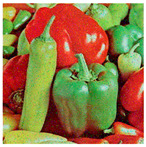	8.0634
10%	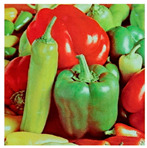	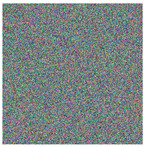	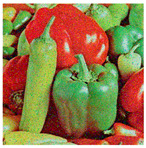	8.0427

**Table 11:** Results from data cut attack.

Data Cut Attack	Original	Cropped Encrypted Image	Decrypted	PSNR
10%	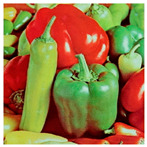	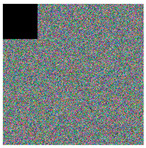	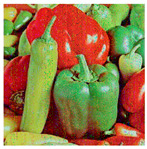	8.0638
25%	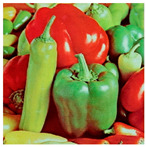	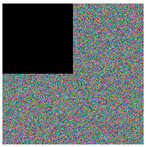	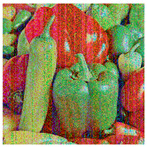	7.9996

**Table 12:** Comparison between different encryption algorithms.

Algorithm	Entropy	Correlation	NPCR	UACI	Key Space
$\mathrm{Proposed}\;(f_{2}$ **)**	7.9993	−0.0002	99.8631	33.5565	10^736^
Hosny et al. [50]	7.9992	0.0044	99.5876	33.4012	10^96^
Chen et al. [51]	7.9960	0.0018	99.6024	33.4883	10^80^
Benaissi et al. [49]	7.9993	0.00204	99.6403	33.5468	10^704^
Gao et al. [52]	7.9993	0.0020	99.6040	33.4538	10^14×3^
Kumar et al. [53]	7.9987	0.0042	99.5600	33.3600	10^153^

## Data Availability

The data presented in this study are available on request from the corresponding author due to future publication purposes.

## References

[1] Makhloufi A.E., Adib S.E., Raissouni N. (2024). Hardware pipelined architecture with reconfigurable key based on the AES algorithm and hamming code for the earth observation satellite application: Sentinel-2 satellite data case. e-Prime-Adv. Electr. Eng. Electron. Energy.

[2] Kumar K., Ramkumar K.R., Kaur A. (2022). A lightweight AES algorithm implementation for encrypting voice messages using field programmable gate arrays. J. King Saud Univ.-Comput. Inf. Sci..

[3] Montano I.H., Diaz J.R., Aranda J.J.G., Molina-Cardín S., López J.J.G., de la Torre Díez I. (2024). Securecipher: An instantaneous synchronization stream encryption system for insider threat data leakage protection. Expert Syst. Appl..

[4] Kumari P., Mondal B. (2024). Lightweight encryption with data and device integrity using NLFSR and PUF for the Internet of Medical Things. Internet Things.

[5] Adeniyi A.E., Jimoh R.G., Awotunde J.B. (2024). A systematic review on elliptic curve cryptography algorithm for internet of things: Categorization, application areas, and security. Comput. Electr. Eng..

[6] Zhang Z., Zhao Y. (2024). Enhanced Elliptic Curve Cryptography (EECC). Procedia Comput. Sci..

[7] Divyashree H.S., Avinash N., Manjunatha B.N., Vishesh J., Mamatha M. (2024). Enhancing secrecy using hybrid elliptic curve cryptography and Diffie Hellman key exchange approach and Young’s double slit experiment optimizer based optimized cross layer in multihop wireless network. Meas. Sens..

[8] Ramakrishna C.J., Reddy D.B.K., Priya B.K., Amritha P.P., Lakshmy K.V. (2024). Analysis of Lightweight Cryptographic Algorithms for IoT Gateways. Procedia Comput. Sci..

[9] Noura H.N., Chehab A. (2022). Efficient binary diffusion matrix structures for dynamic key-dependent cryptographic algorithms. J. Inf. Secur. Appl..

[10] Palmer C. (2023). Quantum cryptography competition yields next-generation standard algorithms. Engineering.

[11] Singamaneni K.K., Muhammad G. (2024). A novel integrated quantum-resistant cryptography for secure scientific data exchange in ad hoc networks. Ad Hoc Networks.

[12] Chawla D., Mehra P.S. (2023). A roadmap from classical cryptography to post-quantum resistant cryptography for 5G-enabled IoT: Challenges, opportunities and solutions. Internet Things.

[13] Jin J., Lei X., Chen C., Li Z. (2024). A fuzzy activation function based zeroing neural network for dynamic Arnold map image cryptography. Math. Comput. Simul..

[14] Gao S., Liu J., Iu H.H.C., Erkan U., Zhou S., Wu R., Tang X. (2024). Development of a Video Encryption Algorithm for Critical Areas Using 2D Extended Schaffer Function Map and Neural Networks. Appl. Math. Model..

[15] Al-Muhammed M.J. (2024). Medical image encryption algorithm based on Fresnel zone formula, differential neural networks, and pixel-guided perturbation techniques. Comput. Electr. Eng..

[16] Wang Y., Su P., Wang Z., Sun J. (2024). Design of double-coupled HR-FN neural network with memristors and its application in image encryption. Appl. Math. Model..

[17] Zhang H., Hu H., Ding W. (2024). VSDHS-CIEA: Color image encryption algorithm based on novel variable-structure discrete hyperchaotic system and cross-plane confusion strategy. Inf. Sci..

[18] Ma W., Li X., Yu T., Wang Z. (2023). A 4D discrete Hopfield neural network-based image encryption scheme with multiple diffusion modes. Optik.

[19] Dhinakaran D., Srinivasan L., Gopalakrishnan S., Anish T.P. (2025). An efficient data mining technique and privacy preservation model for healthcare data using improved darts game optimizer-based weighted deep neural network and hybrid encryption. Biomed. Signal Process. Control.

[20] Saravanaselvan A., Paramasivan B. (2024). An one-time pad cryptographic algorithm with Huffman Source Coding based energy aware sensor node design. Sustain. Comput. Inform. Syst..

[21] Beggas F., Lounici A. (2023). Generation of random sequences using DNA cryptography for OTP encryption. Biosystems.

[22] Basu S., Islam S.H. (2024). Quantum-attack-resilience OTP-based multi-factor mutual authentication and session key agreement scheme for mobile users. Comput. Electr. Eng..

[23] Sheng Q., Fu C., Lin Z., Tie M., Chen J., Sham C.W. (2023). A one-time-pad-like chaotic image encryption scheme using data steganography. J. Inf. Secur. Appl..

[24] Eltoukhy M.M., Khedr A.E., Abdel-Aziz M.M., Hosny K.M. (2023). Robust watermarking method for securing color medical images using Slant-SVD-QFT transforms and OTP encryption. Alex. Eng. J..

[25] Zheng Z. (2022). Shannon Theory. Modern Cryptography 1: A Classical Introduction to Informational and Mathematical Principle.

[26] Elghandour A., Salah A., Karawia A. (2022). A new cryptographic algorithm via a two-dimensional chaotic map. Ain Shams Eng. J..

[27] Liu S., Ye G. (2023). Asymmetric image encryption algorithm using a new chaotic map and an improved radial diffusion. Optik.

[28] Liu X., Tong X., Zhang M., Wang Z. (2024). Constructing of n-dimensional non-degenerate chaotic maps and its application for robust image encryption. Appl. Math. Model..

[29] Ding D., Zhu H., Zhang H., Yang Z., Xie D. (2024). An n-dimensional polynomial modulo chaotic map with controllable range of Lyapunov exponents and its application in color image encryption. Chaos Solitons Fractals.

[30] Munir N., Khan M., Jamal S.S., Hazzazi M.M., Hussain I. (2021). Cryptanalysis of hybrid secure image encryption based on Julia set fractals and three-dimensional Lorenz chaotic map. Math. Comput. Simul..

[31] Xian Y., Wang X. (2021). Fractal sorting matrix and its application on chaotic image encryption. Inf. Sci..

[32] Zhao H., Wang S., Wang S. (2022). Fast image encryption algorithm based on multi-parameter fractal matrix and MPMCML system. Chaos Solitons Fractals.

[33] Gokyildirim A., Çiçek S., Calgan H., Akgul A. (2024). Fractional-order Sprott K chaotic system and its application to biometric iris image encryption. Comput. Biol. Med..

[34] Tong X., Liu X., Zhang M., Wang Z. (2024). A high-quality visual image encryption algorithm utilizing the conservative chaotic system and adaptive embedding. Chaos Solitons Fractals.

[35] Chen X., Hu C. (2017). Adaptive medical image encryption algorithm based on multiple chaotic mapping. Saudi J. Biol. Sci..

[36] Zhu L., Jiang D., Ni J., Wang X., Rong X., Ahmad M. (2022). A visually secure image encryption scheme using adaptive-thresholding sparsification compression sensing model and newly-designed memristive chaotic map. Inf. Sci..

[37] Wen H., Lin Y., Kang S., Zhang X., Zou K. (2024). Secure image encryption algorithm using chaos-based block permutation and weighted bit planes chain diffusion. IScience.

[38] Huo X., Wang X. (2023). Internet of things for smart manufacturing based on advanced encryption standard (AES) algorithm with chaotic system. Results Eng..

[39] Wen H., Xie Z., Wu Z., Lin Y., Feng W. (2024). Exploring the future application of UAVs: Face image privacy protection scheme based on chaos and DNA cryptography. J. King Saud Univ.-Comput. Inf. Sci..

[40] Jin B., Fan L., Zhang B., Lei R., Liu L. (2024). Image encryption hiding algorithm based on digital time-varying delay chaos model and compression sensing technique. Iscience.

[41] Demirkol A.S., Sahin M.E., Karakaya B., Ulutas H., Ascoli A., Tetzlaff R. (2024). Real time hybrid medical image encryption algorithm combining memristor-based chaos with DNA coding. Chaos Solitons Fractals.

[42] Huang Y., Zhang Q., Zhao Y. (2025). Color image encryption algorithm based on hybrid chaos and layered strategies. J. Inf. Secur. Appl..

[43] Klein E., Gross N., Kopelowitz E., Rosenbluh M., Khaykovich L., Kinzel W., Kanter I. (2006). Public-channel cryptography based on mutual chaos pass filters. Phys. Rev..

[44] Coppersmith D. (1994). The Data Encryption Standard (DES) and Its Strength Against Attacks. IBM J. Res. Dev..

[45] Zhang H., Liu D., Wang Z. (2009). Controlling Chaos: Suppression, Synchronization and Chaotification.

[46] Brunovský P. (1968). On the optimal stabilization of nonlinear systems. Czechoslov. Math. J..

[47] Ruppert H., Krug A., Shardt Y.A.W. (2022). Method to design a neural network with minimal number of neurons for approximation problems. IFAC-Pap..

[48] Schimmack M., Mercorelli P. (2022). Anatomy of Chua’s System-Nonlinear Dynamic Electronics for Chaos in the Lab. IFAC-Pap..

[49] Benaissi S., Chikouche N., Hamza R. (2023). A novel image encryption algorithm based on hybrid chaotic maps using a key image. Optik.

[50] Hosny K.M., Kamal S.T., Darwish M.M., Papakostas G.A. (2021). New image encryption algorithm using hyperchaotic system and fibonacci q-matrix. Electronics.

[51] Chen Z., Ye G. (2022). An asymmetric image encryption scheme based on hash SHA-3, RSA and compressive sensing. Optik.

[52] Gao Q., Zhang X. (2024). Multiple-image encryption algorithm based on a new composite chaotic system and 3D coordinate matrix. Chaos Solitons Fractals.

[53] Kumar S., Sharma D. (2024). A chaotic based image encryption scheme using elliptic curve cryptography and genetic algorithm. Artif. Intell. Rev..

